# Examining community perceptions of malaria to inform elimination efforts in Southern Mozambique: a qualitative study

**DOI:** 10.1186/s12936-019-2867-y

**Published:** 2019-07-11

**Authors:** Harvie P. Portugaliza, Beatriz Galatas, Hoticha Nhantumbo, Helder Djive, Ilda Murato, Francisco Saúte, Pedro Aide, Christopher Pell, Khátia Munguambe

**Affiliations:** 1 ISGlobal, Hospital Clínic-Universitat de Barcelona, 08036 Barcelona, Catalonia Spain; 2 Department of Global Health, Amsterdam University Medical Centers, location Academic Medical Center, University of Amsterdam, 1105 AZ Amsterdam, The Netherlands; 3 Department of Biomedical Sciences, Institute of Tropical Medicine, Antwerp, 2000 Belgium; 4 Centro de Investigação em Saúde da Manhiça (CISM), Manhiça, Mozambique; 5 National Institute of Health, Ministry of Health, Maputo, Mozambique; 6 Amsterdam Institute for Global Health and Development AHTC, Tower C4, Paasheuvelweg 25, 1105 BP Amsterdam, The Netherlands; 7 Centre for Social Science and Global Health, University of Amsterdam, Nieuwe Achtergracht 166, 1001 NA Amsterdam, The Netherlands; 8 Universidade Eduardo Mondlane, Maputo, Mozambique

**Keywords:** Community, Elimination, Magude, Malaria, Perceptions, Qualitative

## Abstract

**Background:**

In a background of renewed calls for malaria eradication, several endemic countries in sub-Saharan Africa are contemplating malaria elimination nationally or sub-nationally. In Mozambique, a strategy to eliminate malaria in the south is underway in the context of low endemicity levels and cross-border initiatives to eliminate malaria in South Africa and Eswatini. In this context, a demonstration project aiming to interrupt malaria transmission through mass antimalarial drug administrations and intensified vector control programmes accompanied by community engagement and standard case management was implemented in the Magude District. To ensure the necessary uptake of these interventions, formative qualitative research explored the perceptions, beliefs, attitudes, and practices related to malaria, its prevention and control. The current article describes the results of this study.

**Methods:**

Seventeen focus group discussions were conducted between September and October of 2015 with the community leaders (6), adult men (5), women of reproductive age (5), and traditional healers (1) in Magude prior to the implementation of the project interventions. Respondents discussed perceptions around malaria symptoms, causes, preventions, and treatments.

**Results:**

Knowledge of malaria was linked to awareness of its clinical presentation, and on-going vector control programmes. Perceptions of malaria aetiology were fragmented but related mainly to mosquito-mediated transmission. Reported preventive measures mostly involved mosquito control although participants were aware of the protective limitations of vector control tools. Awareness of asymptomatic carriers and the risk of outdoor malaria transmission were varied. Fever and malaria-like symptoms triggered immediate care-seeking community at health facilities. The identified barriers to malaria treatment included fear/mistrust in Western medicine, distance to health facilities, and lack of transportation.

**Conclusions:**

Several constraints and opportunities will potentially influence malaria elimination in Magude. Malaria awareness, trust in health institutions, and the demand for chemoprophylaxis could facilitate new interventions, such as mass drug administration. A lack of awareness of asymptomatic carriers, inadequate understanding of residual transmission, and barriers to care seeking could jeopardize uptake. Hence, elimination campaigns require strong community engagement and grassroots mobilization.

**Electronic supplementary material:**

The online version of this article (10.1186/s12936-019-2867-y) contains supplementary material, which is available to authorized users.

## Background

In the past decade, the renewed vision of malaria eradication has triggered substantial political attention and financial support in many endemic countries. Malaria eradication is the long-term goal of relieving the world from the burden of malaria through the short-term elimination of disease and transmission in specific regions. To achieve elimination, new malaria intervention packages are needed, as well as technical and economic support to the areas targeting elimination [1, 2]. Although the malaria-related burden of disease remains highest in sub-Saharan Africa (contributing 92% of global cases in 2017) the prospect of elimination has been raised for some previously endemic areas, such as Botswana, South Africa, and Swaziland [3].

According to the 2018 World Malaria Report (WMR) [3], Mozambique ranked 3rd and 8th in terms of number of estimated malaria cases and deaths, respectively. This translates to around 14 thousand deaths and 10 million cases in 2017, with the majority amongst children below the age of five [3]. The high malaria burden in Mozambique results from continuous year-round transmission with a seasonal peak during the rainy period from December to April. Transmission intensity is heterogeneously distributed throughout the country, with higher prevalence estimates in the centre and north (29–68%) compared to the south (2–23%) [4, 5].

Current efforts to prevent and control malaria in Mozambique primarily consist of the nationwide distribution of long-lasting insecticidal bednets (LLINs) through mass campaigns (every 3 years) and routine antenatal care and yearly indoor residual spraying (IRS) in selected districts. Rapid diagnostic tests and artemisinin-based combination therapy (ACT) are implemented as frontline case management measures to avert malaria deaths. Additionally, Intermittent Preventive Treatment of Pregnant Women (IPTp) is offered to address substantial malaria risk in mothers and unborn children [4, 5]. However, concerns have been expressed about the limited coverage and uptake of these programs, i.e., only 16%, 34%, and 88% for bed net, IPTp, and IRS, respectively in 2015 [4, 6, 7]. Meanwhile, malaria cases are increasing whereas funding for national malaria prevention and control has stagnated over the last 5 years [3].

Despite the concerns of increasing malaria incidence, the National Malaria Control Programme (NMCP) has identified southern Mozambique as a potential target area for elimination [4, 5]. To support the design of the NMCP’s malaria elimination plan for southern Mozambique, a malaria elimination project was implemented in the district of Magude [8]. This project aimed to interrupt malaria transmission through mass antimalarial drug administration (MDA) and vector control (IRS and LLINs) combined with a campaign of strong community engagement and standard case management.

The effectiveness of these interventions depends significantly on achieving high coverage levels (at least 80% for MDA, 85% for IRS, and 100% for LLINs [9–11]) in target communities. This can be a challenge, particularly in the case of MDA, which requires high uptake among all members of a community regardless of infections status [12]. Achieving high MDA coverage requires the design of tailored interventions combined with intense community engagement activities according to local contexts and understandings of malaria [13, 14].

To inform the design of the elimination strategy, and the accompanying community engagement in Magude, formative qualitative research was conducted to examine local understandings of malaria and its prevention and control prior to the deployment of interventions. This article describes community members’ reported malaria-related perceptions, beliefs, attitudes, and practices, and examines their relevance for the design of malaria elimination strategies.

## Methods

### Study area

Magude District, in northwestern Maputo Province, southern Mozambique, borders South Africa’s Kruger National Park. Magude covers 6961 km^2^ with five administrative posts, and is home to approximately 50,000 residents spread across 11,000 family compounds. Agriculture and fishing are the main livelihood activities. Magude has open forests, savannahs, and one permanent river (*Incomati*). The majority of houses are traditional round or rectangular huts with walls constructed mainly using cane, cement, and mud bricks. More than 80% of the households count on either a traditional latrine or no form of sanitation facility. In 2015, Magude counted with seven rural health centres (but no district hospital), which are all located near main roads in its respective administrative posts. The median distance from households to the nearest health facility is 2.7 km (interquartile range [IQR] 1.4–7.9 km), although households can be as close as 15 m or as far as 38.8 km from the health facility. The far-flung areas within this district are covered by 27 community health workers or *Agentes Polivalentes Elementares* (APEs) who provide basic healthcare services and referrals to the closest health centre. The average distance between households and the assigned APE is around 6.3 km (median of 4.4 km, IQR 2.8–5.4 km). According to the guidelines of Mozambique’s Community Health Programme, one APE is expected to cover 500 to 1200 inhabitants [4, 5]. However, to our knowledge, there has been no assessment on the actual APE coverage in Magude district. Malaria has historically been the main cause of morbidity in the district, with a yearly incidence higher than 200 cases per 1000 (Galatas et al. pers. commun.).

### Study design

A qualitative study examined community perceptions of malaria in the district of Magude prior to the implementation of an intervention package aiming to interrupt malaria transmission in the area. This study used a grounded theory approach to generate and understand emergent theories and patterns about the local perceptions of malaria and leaned towards post-positivism by considering participants’ perspectives around malaria-related concepts while maintaining objectivity (guided by the biomedical explanatory models) and awareness of potential biases [15].

### Sampling strategy

Participants were selected to elicit the opinions and capture the lay perspectives of MDA recipients as well as household and community-level decision-makers. Purposive sampling was performed to achieve uniform representation of community groups and administrative locations through consultations with community key informants. Key informants acted as an intermediary between the research team and their communities, and supported the identification and mobilization of participants.

### Data collection

A semi-structured focus group discussion (FGD) guide was designed to capture perceptions of malaria and its related concepts (i.e., symptoms, causes, controls, and treatments). The guide questions were prepared in Portuguese, and pilot tested in the local language Changana. After subsequent refinements, all FGDs were conducted in Changana. The interviewers, who are all fluent in Portuguese and Changana, were trained to facilitate the FGDs. FGDs were carried out between September and October 2015, with community leaders, traditional healers, adult men (≥ 18 years old), and women of reproductive age (15–49 years old) in all administrative posts until saturation was reached, i.e., no new additional information emerged.

### Data analysis

FGDs were audio-recorded, transcribed, and translated from Changana into Portuguese and then into English by certified local translators. Transcripts were initially read and examined for themes relevant to the research question. Transcripts were then imported into NVivo 12 software (QSR International) for in-depth content analysis. The analysis approach was mainly inductive, with flexibility in identifying relevant themes. Topics that showed within- and between-group consensus, divergence, and uniqueness were further examined. Finally, the themes were critically discussed until researchers reached agreement on their relevance and the patterns observed across the different respondent groups.

## Results

In total, 17 FGDs were conducted with community leaders comprising village chiefs, traditional leaders, and heads of large families (6); men older than 18 years-old (5); women of reproductive age (15–49 years-old) (5); and, traditional healers from the Association of Traditional Healers of Mozambique (AMETRAMO) (1). The number of participants per FGD ranged from three to 12 (Additional file 1).

### Demographic characteristics

Table 1 presents the demographics of participants of each community group represented in the FGDs. Community leaders comprised mainly of male participants aged 30–83 years (median 63). Traditional healers were mainly female aged 21–51 years (median 30). The age of men and women groups ranged between 18 and 76 years (median 26) and 15–60 years (median 30), respectively. Most participants were married or living in marital union, had a low level of education (mostly primary level), and worked as farmer or charcoal producers. Participants were largely Christian, but a few identified themselves as Atheists, Muslims, Animists, and Zionists.

**Table 1:** Demographics of participants per community group

Variables	Community leaders	Men	Women	Healer
Gender				
Male	94.2% (49/52)	100% (39/39)	0 (0/53)	10% (1/10)
Female	5.7% (3/52)	0 (0/39)	100% (53/53)	90% (9/10)
Age (years)				
Range	30–83	18–76	15–60	21–51
Median	63	28	30	30
Mean	59	31	31	32
Marital status				
Single	1.9% (1/52)	30.8% (12/39)	35.8% (19/53)	40% (4/10)
Married	11.5% (6/52)	2.6% (1/39)	0 (0/53)	10% (1/10)
União (Marital Union)^b^	81% (42/52)	66.7% (26/39)	51.8% (28/53)	50% (5/10)
Widowhood	5.8% (3/52)	0 (0/39)	11.3% (6/53)	0 (0/10)
Education				
None	28.9% (13/45)	5.1% (2/39)	20.8% (11/53)	10% (1/10)
Primary	57.8% (26/45)	46.2% (18/39)	37.7% (20/53)	80% (8/10)
Secondary	11.1% (5/45)	43.6% (17/39)	41.5% (22/53)	10% (1/10)
Tertiary	2.2% (1/45)	5.1% (2/39)	0 (0/53)	0 (0/10)
Religion				
Christianity	59.6% (31/52)	74.4% (29/39)	71.2% (38/53)	0 (0/10)
Atheism	17.3% (9/52)	23.1 (9/39)	15.1% (8/53)	100% (10/10)
Animism	19.2 (10/52)	2.6 (1/39)	5.7% (3/53)	0 (0/10)
Islam	3.8% (2/52)	0 (0/39)	0 (0/53)	0 (0/10)
Zionism	0 (0/52)	0 (0/39)	7.5% (4/53)	0 (0/10)
Occupation				
Farmer	63.5% (33/52)	20.5.7% (8/39)	54.7% (29/53)	0 (0/10)
Coal producer	0 (0/52)	23.1% (9/39)	0 (0/53)	0 (0/10)
Trad. Healer	0 (0/52)	0 (0/39)	0 (0/53)	100% (10/10)
Home-based^a^	1.9% (1/52)	15.4% (6/39)	32.1% (17/53)	0 (0/10)
Salesperson	7.7% (4/52)	7.7% (3/39)	1.9% (1/53)	0 (0/10)
Service/laborer	3.8% (2/52)	7.7% (3/39)	5.6% (3/53)	0 (0/10)
Driver	1.9% (1/52)	5.1% (2/39)	0 (0/53)	0 (0/10)
Teacher	1.9% (1/52)	2.6% (1/39)	0 (0/53)	0 (0/10)
Student	0 (0/52)	2.6% (1/39)	3.8% (2/53)	0 (0/10)
Others	19.2 (10/52)	15.4% (6/39)	1.9% (1/53)	0 (0/10)
Total	52	39	53	10

^a^ *Housewives/househusbands*

^b^União: Marital union without marriage certificate

### Perceptions of malaria and its symptoms in Magude

In the local dialect, Changana, the collective symptoms of malaria were referred to as the traditional illness *Mututumelo* (syn. *Muzototo, Mututumela*) or *Dzedzedze* (syn. *Madzedzedze*). ‘Malaria’ connoted a diagnosis made by the healthcare provider after presenting with signs traditionally linked to *Mututumelo* or *Dzedzedze*. Participants believed that these terms represented a single disease on the basis of symptoms or after a diagnosis. Hence, in the following quotation (and all others), ‘malaria’ is used to describe syndromes that may or may not overlap with the biomedical definition.

*It’s the same thing… when you go to the hospital they say you have malaria. Traditionally they say that you have Muzototo.*

FGD4_HA10 adult men

*Dzedzede is the same as malaria…because Dzedzedze is when you do not feel the body being good and feeling cold.*

FGD5_MIR13 women of reproductive age

For respondents, ‘malaria’ was a disease that causes suffering among children and adults. Awareness of malaria and the perceived symptoms was noticeably high in all groups. Participants identified fever, cold, joint pain, headache, and vomiting (*caguba*) as typical symptoms. Weakness, body pain, back pain, stomachache, lack of appetite, shivering and trembling were also mentioned.

*What happens now is that you just feel headaches*, *feel body aches and cold, it´s malaria*, *caused by mosquito.*

FGD1_MT17 Traditional healers

Perceived symptoms of childhood malaria were convulsions, abdominal swelling, stomachache, fever, and weakness.

*I have been teaching the population about malaria…I met along the way two people going to the healers with a child who was convulsed with a swollen belly. I told them to go to the hospital [for malaria treatment] before going to the healers.* FGD2_LC3 community leaders

### Perceived forms of malaria

Two forms of malaria illness emerged from the FGDs. One involved signs of unusual behaviour or confused thinking. Another was termed “bile” and, especially female respondents, described as vomiting of greenish fluid accompanied by other malaria symptoms, such as weakness and shivering.

*Malaria goes up to the head [it gets worse] and you start talking nonsense… you start having problems with the neighbors while you’re not doing it on purpose… it’s malaria that went up, so this is not a good [form] of malaria… the only way is to go to the hospital.*

FGD1_LC1 community leaders

*I was saying it was Mututumelo (malaria) and if we vomited they said it was bile.*

FGD4_MIR12 women of reproductive age

Fever or “heating up of body” was described consistently as a symptom that accompanied malaria illness and characterized by an increase in body temperature. It was interpreted as indication that an immediate ‘hospital’ visit was needed. ‘Fever’ sometimes was used as a generic term for a group of symptoms that resembled malaria. ‘Malaria’ and ‘fever’ were not however viewed as being the same, but rather distinguished through a malaria test and medication, such as ‘paracetamol’.

*When the person is in this [malaria] situation, the body heats up a lot with fevers…we first wet a cloth, put it in the armpits, on the neck and the feet to be able to take him or her quickly to the hospital.*

FGD2_HA4 adult men

*[Fever and Malaria] is not the same thing because I can tremble, have headaches and vomiting but when I take paracetamol it stops. While in malaria, I can take paracetamol from 1 to 30 [tablets] and I still feel hot …it will not pass without first taking malaria pills.*

FGD1_MIR6 women of reproductive age

Few participants attributed some of the malaria symptoms to cholera, tuberculosis, and HIV/AIDS. They emphasized that test confirmation from the healthcare provider is needed to diagnose malaria.

### Perceived causes of malaria

Participants identified mosquitoes as the main cause of malaria and perceived transmission as occurring through direct and indirect pathways. Malaria-causing mosquitoes were reportedly female with habitats that included water, animals, and plants. Women reported poor hygiene and sanitation as the reason for the presence of mosquitoes. And respondents viewed mosquitoes and water as vital factors for the occurrence of malaria illness in the community. Mosquitoes were viewed as abundant and likely to bite during the rainy season, close to ditches, streams, and boreholes; or generally in the presence of dirty and stagnant waters.

*[Bouts of malaria] are caused by mosquitoes that stay in the water where they [live] and reproduce. They come out and bite people inside the houses… if [people] do not close [the windows] the mosquitoes appear inside the houses and bite people.*

FGD5_LC14 community leader

Respondents mentioned that animals played a role in malaria transmission, either as carriers of the disease or as enhancers of mosquito numbers. ‘Malaria’ was viewed as caused by an agent that came from animals and transferred to human through mosquito bites and animal waste (e.g., urine). In other scenarios, the presence of livestock near houses attracted malaria-causing mosquitoes to enter nearby households.

*If someone starts getting sick near the barn, a mosquito bites an animal and then it bites me. I end up getting malaria.*

FGD3_HA7 adult men

Malaria illness was also linked to the consumption of fruit and vegetables. Also, participants identified unpruned trees, grasses, and areas near plantations as the lairs of malaria-causing mosquitoes.

Malaria parasites (e.g., *Plasmodium falciparum*) were not mentioned explicitly as the causative agent of the disease. However, participants hinted at an additional component necessary for malaria illness to occur that involved transfer of biological materials carried by mosquitoes. These materials were mainly infected blood, fecal matter, urine, and dirt. Less frequently, they mentioned ‘virus’, ‘poison’, and ‘venom’.

*In another case, when [a mosquito] bites someone with malaria and as soon as it bites me, I will get the disease. The blood of that person comes to infect mine.*

FGD3_HA7 adult men

Flies and various insects were also identified as carriers of dirt that causes malaria, especially among children. In addition, bad-smelling, witchcraft, and sorcery were mentioned as perceived causes of malaria.

*Those big [flies] that go into those latrines because we don’t have improved latrines there… the person digs a pit and [defecates]. Those flies can get out of there and they will land on the food and the children eat that food and contract malaria.*

FGD1_LC1 community leaders

### Prevention of malaria transmission

Preventive measures reported by the community against malaria transmission could be systematically divided into three main groups: (1) mosquito avoidance, (2) chemoprophylaxis, and (3) other measures of ‘malaria’ prevention.

#### Mosquito avoidance

Mosquito avoidance encompassed practices linked to mosquito-human contact evasion and actions that target places or objects associated with mosquito breeding and resting sites (although these two concepts were often intertwined). Using a bed net while sleeping emerged as the most common practice to avoid mosquito bites. Bed net ownership indicated an assurance to reduce chances of getting malaria. In fact, women underscored bed net as the only option to avoid malaria.

*[To avoid malaria] let’s sleep under ‘Mutchiquitelo’ [mosquito net]. There is nothing else, it’s just ‘mutchiquitelo’… you do nothing else, just stretch [the mosquito net].*

FGD2_MIR2 women of reproductive age

*Keep the spaces clean and use mosquito nets. When you go to sleep, stretch the net and sleep inside it so that the mosquito does not bite you.*

FGD3_HA7 adult men

*It’s the mosquito net, it’s necessary in the afternoon when it’s already dark, to stretch the mosquito net, to close it here in bed, so that the mosquito does not enter, or if there is a small child, the child must be there before we sleep while talking. So when we get back, we, husband and wife, are going to get in there and close it.*

FGD3_LC8 community leaders

On the other hand, several respondents described bed net non-use and misuse. Community leaders reported that many of the bed nets distributed in the past were still ‘in the package’ and unused by the community. The non-use of bed nets was explained in terms of discomfort while sleeping. Participants also reported that bed nets were used as fishing nets and/or to protect crops.

*The mosquito nets that we talked about, so far most of it are still in the plastic and they have not been used yet.*

FGD2_LC3 community leaders

*We use a mosquito net but you find people in those places where they were told to use a mosquito net… [They] received a mosquito net and heard that they have to sleep inside it, and reply yes. When you leave and they take it (bed net) home and say that they cannot sleep in a mosquito net, some use it for fishing.*

FGD4_HA10 adult men

Other methods of mosquito avoidance included closing windows and using insect repellants or coils. Also, participants perceived that cleanliness or improving hygiene and sanitation conditions will repel malaria-causing mosquitoes.

*In our houses, when the night falls we need to close the windows to avoid mosquitoes from entering. We need to clean and cover the pits with water, so you can prevent yourself from having malaria by avoiding mosquitoes from landing.*

FGD5_LC14 community leaders

*When I am seated if it’s not sprayed, I stay in the room and I buy that coil that is burned, how is it called? It’s called dragon. I burn it because it kills the mosquito for sure… you won’t get bitten by mosquito when you’re chatting. You cannot chat within a mosquito net. You can just chat in the seating room.*

FGD1_LC1 community leaders

In contrast to the revealed knowledge about mosquito avoidance, their discourse on actual practices suggests that people open the windows at night, sit in communal areas during biting hours, sleep late, and do not use insect repellants or insecticidal coils. Women participants acknowledged the importance of mosquito repellants to avoid mosquito bites outside bed net protection zones. However, they reported to never or rarely use protective methods aside from bed net due to lack of purchasing capacity.

*We need to use a mosquito net… but even in the day the mosquito bites you. Even when you are seated they can come and bite you, they can bite you before sleeping in the net.*

FGD5_MIR13 women of reproductive age

Actions that target places or objects associated with mosquito breeding and resting sites were described as a fundamental control measure to reduce malaria-causing mosquitoes. Most practices described for breeding site elimination included disposal of stagnant water or dirty water and containers that capture rainwater. Adult men also mentioned the treatment of water bodies near houses to eliminate mosquitoes.

*We need to clean our homes because there are places where we store water, the bowls keep rainwater… It happens that mosquitoes live in those places.*

FGD1_MT17 traditional healers

*Get rid of bottles that usually retain water because the mosquitoes usually live in waters inside the cafulo (coconut shell).*

FGD1_LC1 community leaders

*The water has to be far from houses. Inside houses there can be holes with water where mosquitoes lay eggs and reproduce.*

FGD5_MIR13 women of reproductive age

*There is tlhive, what we call a pond, I do not know how to say but, the great help for those tlhives that we call lagoons, if we have medicine to put there, maybe the mosquito can disappear because that water is too much and you cannot cover the tlhive. We are close to it, we build close to it, we will not be able to cover it.*

FGD3_HA7 adult men

Additional practices targeting mosquitoes comprised the acceptance of IRS, removal of garbage or clutter, pruning plants, and avoidance of animal pens near houses.

*We have to accept [IRS] at home. Do not refuse so that we can scare away that existing mosquitoes.*

FGD1_MT17 Traditional healers

*We have to avoid hoarding garbage in our homes. We take care of the trash because it will cause us mosquitoes… malaria. Malaria comes from mosquitoes.*

FGD2_HA4 adult men

*At home, we need to take care of ourselves, clean the house and clear the grass near the house, leave the space clean so that the mosquito cannot find a place to stay.*

FGD3_HA7 community leaders

*[To prevent malaria], lessen the nearby shrubs and prune the trees.*

FGD1_MIR6 women of reproductive age

However, participants also reported refusals to IRS. Community leaders described IRS as triggering cough, promoting cockroach infestation, and making houses dirty. Aside from the perceived side effects, those with concrete houses and air-conditioned rooms refused IRS because of the apparent absence of mosquitoes.

*They will accept mosquito nets but not spraying because it causes coughing.*

FGD2_LC3 community leaders

*It’s the younger ones who deny [IRS] and will say this brings cockroaches and makes houses dirty.*

FGD3_LC8 community leaders

#### Chemoprophylaxis

Chemoprophylaxis emerged as a hypothetical solution in the form of protective pills that participants reported would avoid mosquito bites and initiation of malaria symptoms. Respondents were aware of the concept of malaria chemoprophylaxis as a result of the community engagement campaigns that were already underway during the study period, as well as through their experience in South Africa where malaria prophylaxis is offered to travelers going to or coming from endemic areas.

*I can spray the house, sleep under the mosquito net, but many of us study at night class. At school there are no mosquito nets and it has not been sprayed, there the person is bitten and can get malaria, whereas if there is a pill that the hospital has created that protects us, the mosquito can bite but it will not do anything.*

FGD1_MIR6 women of reproductive age

*For the prevention of this disease, malaria, it is necessary that the government brings us…tablets to take. Similar to South Africa…there are tablets that have been distributed and people have taken and got the body drugged so that when the mosquito bites, they do not get sick.*

FGD3_HA7 adult men

During the FGDs, women did not mention IPTp as a form of malaria prevention. However, a few adult men hinted that the hospital offered IPTp and a community leader identified *Fansidar* (Sulfadoxine–Pyrimethamine) as an antimalarial drug, although he did not associate this drug to IPTp.

*[Pregnant women] must go to the hospital. There are what they give [pregnant women] to prevent this evil that comes from the mosquito.*

FGD2_HA4 adult men

*In the hospital when they test you and confirm Malaria, they give you a number of pills to be taken… of which I believe I’m right in recalling right now, that are called Fansidar.*

FGD1_LC1 community leaders

#### Other measures of ‘malaria’ prevention

Few participants mentioned practices that they believed would prevent malaria illness. These practices were handwashing, drinking clean water, cleaning the air, and practices related to avoidance of fecal contamination.

*To prevent malaria, it’s necessary to have the residences cleaned up. When the children go to defecate…quickly go clean the feces… it reduces [malaria]. That’s what makes …no illnesses in that household.*

FGD1_LC5 community leaders

*Clean up the house and keep it always neat, because the air we breathe can also create diseases here inside the house.*

FGD1_LC5 community leaders

### Perceived risk of residual malaria transmission

Participants acknowledged that the existing malaria control program targeting mosquitoes offers limited protection against residual transmission. For example, free bed net distribution and yearly IRS were perceived as not protecting them all the time, hence the demand for a new intervention that they called “the pills that protect”.

*How did I get sick while the house was sprayed? I sleep inside the mosquito net… this remedy doesn’t work then. While [IRS and bed net] work… but the mosquito has bitten you when you were outside. So when I hear this opinion of the pills, I’m happy because I’m already protected, so that’s what I say, the main cause is the mosquito.*

FGD1_LC1 community leaders

In addition, contradictory notions emerged among participants when expressing concepts of residual malaria transmission or outdoor mosquito biting. Respondents described the indoor home environment as mosquito feeding ground where biting occurs and the outdoor environment as their lair and site of reproduction. While this general concept may indicate low awareness of outdoor transmission, other groups, especially women, showed high awareness on the risk of getting malaria outdoor.

*Many of us study at night class. At school there are no mosquito nets and it has not been sprayed, there the person is bitten and can get malaria.*

FGD1_MIR6 women of reproductive age

Overall, participants identified several activities that they believed could put people at risk of malaria infection outdoors but not necessarily facilitated by mosquito biting. These included harvesting vegetables or fruits, working in cane plantation, working with livestock, going to the forest, defecating in the bush, fetching water in the river, washing clothes in the river, walking near dumpsites, and chatting in the backyard.

### Perception of malaria case management

Respondents described how suspected patients who accessed the health facility normally received a rapid diagnostic test (RDT) to be appropriately treated against malaria, which was generally well accepted. Malaria treatments were viewed as measures to prevent the progression of malaria symptoms and averting death. The perceived treatment options could be divided into home remedy, western medicine, and traditional medicine. Home remedy implied self-treatment or practices to abate fever. Participants reported cooling the body with wet clothes and taking ‘paracetamol’ to control fever.

*The body heats up a lot, with fevers, we first wet a cloth, put it in the armpits, put it on the neck and the feet to be able to take him or her quickly to the hospital, we have no other way of prevention*.

FGD2_HA4 adult men

Participants argued that malaria can only be cured by medicines prescribed by healthcare providers wherein malaria case management included diagnosis through RDT and artemisinin-based combination therapy (ACT). A few participants were aware of the anti-malarial drugs used in the health facility by mentioning Coartem (Artemether–Lumefantrine).

*While in the hospital they make analysis and discover the number of crosses (parasite density) that indicates that you have malaria. Then they give the medicine to take the way they tell you.*

FGD4_LC11 community leaders

*When a child or an adult has fever…take the person to the hospital to be tested, what they call lab tests, in which they make a small cut in the finger to test, to check out if he has the Malaria virus, there is no other thing, in my opinion.*

FDG1_LC1 community leaders

*For the treatment of malaria it is necessary that when the person feels any of these symptoms, such as pains in the joints, lack of appetite and [fever], you have to go to the hospital to do malaria test. If the disease is found they give you Coartem to take.*

FGD4_HA10 adult men

In contrast, several community members, especially older people, feared and doubted western medicines. This arose from perceived drug-related side effects. They particularly recalled drug side effects after taking tablets from a population-wide deworming campaign.

*There were tablets that we have taken for some time and after consuming the tablets some people got dizzy, and some people did not clarify anything about the side effects.*

FGD4_HA10 adult men

*Another one when taking tablet, says that it does not pass here (indicating the throat).*

—FGD4_LC11 community leaders

Participants from Mahele (a remote administrative post/locality on the northwestern part of Magude) described that locals who do not visit the hospital tend to self-medicate using traditional (non-biomedical) medicine. Such medicines, in the form of plant mixture prepared in a clay pot (*ximbitane*), were used to prevent and treat diseases such as malaria and measles. However, respondents reported that traditional medication was ineffective against malaria. Participants, even traditional healers, reported that malaria has never been and can never be cured by traditional medicines since it’s a “non-traditional disease”.

*In the old days, every year we take a mixture of plants called Mpondzo to avoid diseases that are still to come. Also, if you have the habit of eating kakana (Momordica balsamina) without putting peanuts, it is because you have something that you want to avoid. There’s a disease we call Xitsinana (measles)… What’s the name? Sarampo (measles)!*

FGD4_HA10 adult men

*There is a tree that most people said had a positive effect on the cure of malaria. I saw several times in Namaacha when someone has malaria, boils it and drinks. It was healing [them], but lately it was no longer used. We now use CoArtem… In Namaacha, when people wander in the hills, there were many of these trees. Many people from the hidden areas never had the habit of going to the hospital, people self*-*medicated* via *traditional medicine.*

FGD4_HA10 adult men

*Traditional remedies do not cure, because this is not a traditional disease, it doesn’t combine with roots, it needs pills*.

FGD1_LC5 community leaders

**Fig. 1:**
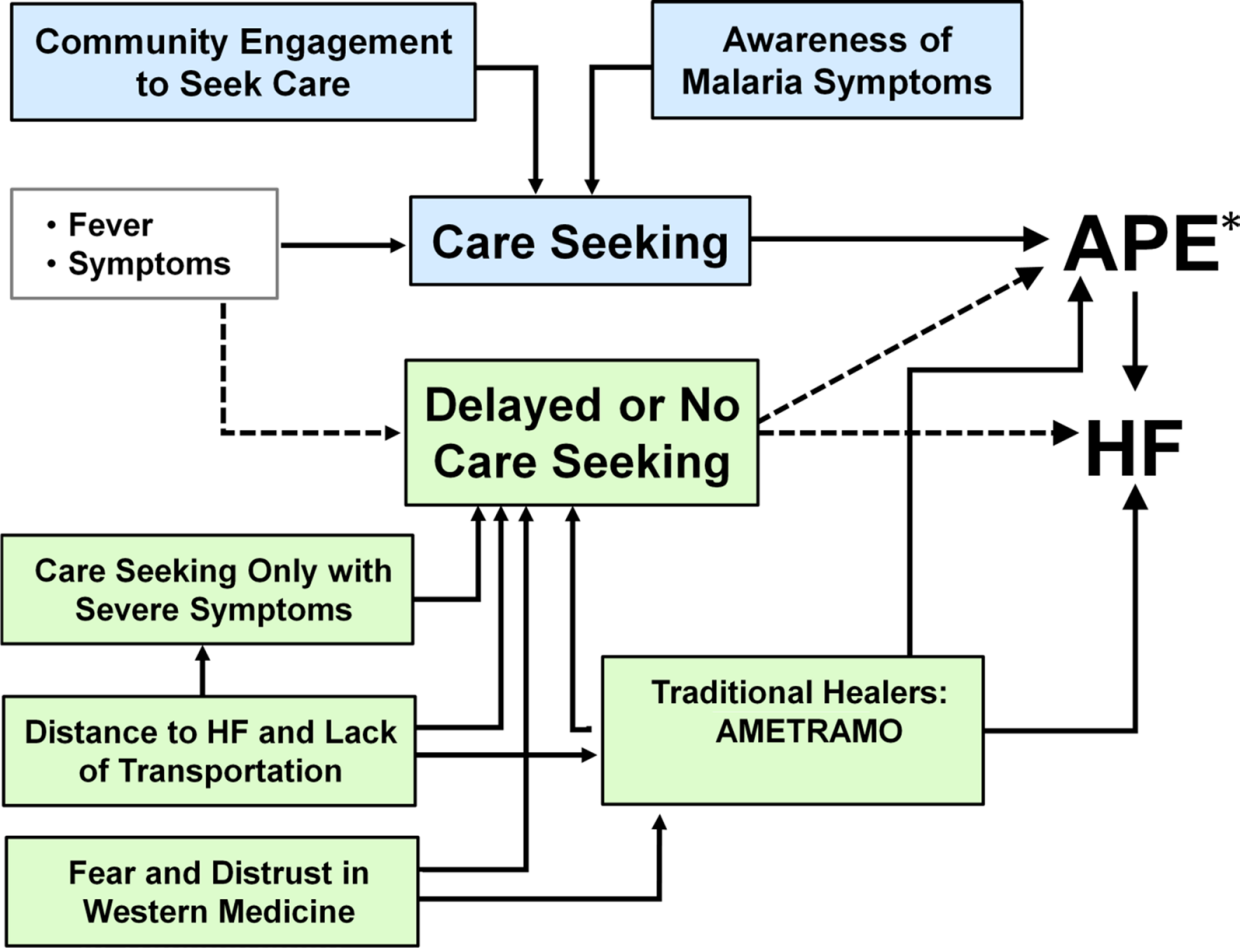
Facilitators and barriers to the early phase of malaria treatment. *APE* Agentes Polivalentes Elementares (Community Health Workers), *HF* Health Facility. *APEs can also be the end of the care seeking chain if symptoms are not severe

Several barriers were reported to accessing health facilities for malaria treatment in Magude District (Fig. 1). Participants described that the long distance from their houses to health facilities and the inaccessible roads delayed—or completely prevented—them from seeking malaria treatment. The figure of the Community Health Workers or *Agentes Polivalentes Elementares* (APE) who act as a bridge between the community and the health facility appeared to provide a temporary solution.

*For me who lives in Makunanine [Magude Sede administrative post], the hospital is far away, but there are [health] post agents. I will go to one of them, he is always available because it can be at night, and the disease won’t wait for the morning. You go and get there before taking pills, because once you take the pills, the agent won’t diagnose you, even if he tests you nothing will be detected. They do the tests and if you are malaria infected, he starts giving you pills, if you see that you won’t make it, he will give me a document that says I went to see him and then I came to the hospital.*

FGD1_LC1 community leaders

*The government has trained people who have the responsibility of helping the population in different places. If the leader has the phone number of the APE, if someone does not feel well, he or she can call the APE for help. The APE must monitor and count with enough tablets to help. If he cannot, the APE can write a letter and send the person to the hospital (referral health centre).*

FGD3_HA7 adult men

Community members from remote areas only reported seeking medical help when disease progressed to severe condition. This delayed health-seeking behaviour appeared to be associated with distance to health facility and a lack of information.

*People here when they do not feel well, are in the habit of going to the forest to look for roots to boil, to take and to feel better. When you talk about taking pills it’s difficult. He needs to be serious so he can go to the hospital if he feels that he cannot do anything even open a pit to put their rubbish or do anything else. It’s only in this state that he agrees to go to the hospital.*

FGD2_LC3 community leaders

## Discussion

FGDs prior to the initiation of the malaria elimination project in Magude, indicate that the community was aware of malaria, trusted formal healthcare institutions, and identified gaps in prevention provided by the available vector control tools. However, it also reveals limited knowledge and misunderstandings about the concepts of malaria transmission and preventive measures. Additional significant findings include the varied awareness about asymptomatic carriers, the risk of outdoor malaria transmission, and the openness of the participants to new interventions. The possible basis to these notions and their implication to the on-going malaria elimination campaign are discussed below.

In Magude, malaria remains a prevalent disease with around 195 cases per 1000 inhabitants in 2014 [16]. The area has seen malaria prevention and control initiatives combined with Information, Education, and Communication (IEC) campaigns [4, 8]. These efforts are reflected in the respondents’ awareness of malaria and its prevention and offer promise for planned elimination campaigns because, as in other endemic settings, malaria knowledge and awareness promotes case reporting and care seeking when a malaria-like symptom arises [17, 18]. For community members, the ambiguousness of malaria symptoms (e.g., fever vs malaria) can pose serious challenges to prevention and control [19, 20]. In contrast, respondents from Magude viewed ‘fever’ and ‘malaria’ as separate phenomena, with the latter encompassing the former. Fever alone could trigger care-seeking at health facility or to an APE. In addition, two distinct symptoms were associated with malaria and bouts of severe disease that also led to care-seeking: ‘confused thinking’ is likely to describe delirium; whereas “bile” may imply persistent vomiting, which is particularly common in children with severe malaria [21, 22].

The high awareness of malaria symptomatology and care-seeking could have the following implications for the transition to elimination. First, the implementation of MDAs and vector control in Magude is expected to drastically reduce transmission in a short period of time [23], after which reactive interventions will be implemented to sustain the gains. These reactions will likely be triggered by malaria infections that are passively detected at health facilities or by the APE, and its success is highly dependent on the level of awareness of clinical malaria presentation and will to seek care at community level. Second, as transmission decreases, communities should be informed of the existence of other causes of fever; and febrile patients should be screened for alternative sources of fever such as viral infections [24] and pneumonia [25].

Few participants alluded to the concept of sub-clinical infections that can still act as sources of transmission. This has been previously reported in other areas where villagers seemed unaware and having difficulties understanding the concept of asymptomatic carriers [26]. This lack of awareness of the existence of asymptomatic infection reinforces the need to deliver specific messages about the concept and importance of symptomless infections prior to the implementation of interventions, such as MDA, which target this parasite reservoir at a population level [27]. However, this study also revealed that the community is aware of the protection gaps around vector control tools, and seemed to welcome chemoprophylaxis as an additional prevention measure. A thorough review of the acceptability to MDAs and its potential barriers in Magude will be presented in a separate publication.

In contrast to their familiarity with the symptoms of malaria, respondents’ awareness of malaria aetiology is not comprehensive and fragmented with concepts extrapolated from vector control programmes. Perceived causes of malaria infection were diverse with many underlying agents, including animals and plants, but generally mosquito habitats and behaviour. In Mozambique, *Anopheles arabiensis* and *Anopheles funestus* are the primary vectors of malaria with tendencies to feed on animals and humans indoors and outdoors [28, 29]. Of all the perceived causes, mosquitoes and water emerged as vital causal elements for malaria infection. These responses reflect the extensive efforts at malaria prevention and control through vector control programmes (i.e., IRS and bed net distribution). However, this also suggests that the IEC that accompanied these (and other) programmes did not effectively communicate the role of anopheline mosquito in transmitting the parasite. For instance, several reported measures are not technically linked to malaria prevention, and vector-directed practices may not be specific to anopheline mosquito behaviour. Identifying the mosquito as the direct causative agent of malaria could represent a challenge when promoting preventive interventions that do not directly target mosquitoes.

In Magude, the existing malaria-risk avoiding practices focused mainly on mosquito avoidance, which includes bed net use, IRS, and the elimination of mosquito breeding and resting sites. Nationwide, from 2007 to 2015, household bed net ownership ranged from 16 to 66%, with reported usage between 7 and 52% among pregnant women and children under 5 years of age [4, 5]. Respondents described their reasons for using bed nets as being based on a desire to protect themselves from malaria rather than avoiding nuisance bites, as reported elsewhere [30]. Anecdotal reports of misusing bed nets into a livelihood tool (e.g., fishing net) reiterate how poverty hinders the full potential of malaria control [31, 32]. FGD participants reported accepting IRS because of strong community leadership and general compliance with the ‘law’—a socially constructed concept present in Mozambique [33, 34]. But, as in the neighboring district of Manhiça, acceptance was affected by rumored side effects and socioeconomic status [33].

Some participants, particularly women, listed activities in which outdoor mosquito-human contact took place, and in a few instances associated these activities to the risk of transmission. Similarly, the UNFPA [35] reported that drought in Maputo Province resulted in new and prolonged outdoor activities among women, who tend to gather woods and reed plants for daily subsistence, and may spend 10 to 12 h outside, including at dawn and during the night, to collect water. Despite insufficient evidence from the areas to quantify ongoing malaria transmission outdoors, these findings highlight the potential limitations of vector control interventions that focus on the indoor environment and open the space for tools targeting residual forms of transmission. Positive opinions about targeting mosquito breeding sites (e.g., treating water bodies) could indicate openness to try new vector control tools, such as larval source management.

IPTp was not mentioned by respondents. This reflects the generally low IPTp awareness and uptake throughout Mozambique (16–43%) [4, 5] and the need for focused messages to address this gap. Low levels of IPTp uptake has been explained in terms of the complexities of antenatal care (including delayed first visits, negative attitudes of health professionals towards the intervention, and lack of supporting infrastructure), household decision making, perceived drug side effects, poor awareness, and lack of education, among others [6, 36, 37]. Considering the emergent positive views on protective pills captured through this study as a result of the ongoing IEC to promote MDA participation, the low level of awareness of IPTp coverage may be associated with insufficient messaging to the community about the functionality and availability of this chemoprevention measure.

Malaria treatment means abating symptoms and averting deaths through western medicine or healthcare providers. In Magude, visible malaria programs and related community engagement positively influence the decision to seek and access treatment but structural factors constrained the early phase of the treatment pathway. In particular, transportation and distance delay malaria treatment while efforts in installing localized APEs offer a relative solution to the multifaceted problem. The traditional approach to treating malaria was described as rarely taken, even by some traditional healers. This finding conflicts with that of many other studies, which have highlighted traditional medicine as the first source of assistance for malaria treatment in sub-Saharan Africa [38]. Mozambican policy towards licensing traditional healers through the *Associação de Médicos Tradicionais de Moçambique* (AMETRAMO) [39] might have driven inclusivity of healers into different malaria-related campaigns, which in effect influence their beliefs about malaria. This way, traditional healers constructively contribute to malaria control and case referral. On the other hand, healers’ perception towards malaria treatment should be interpreted cautiously due to overrepresentation and potential desirability bias of trained healers and underrepresentation of sorcerers or healers who are not part of AMETRAMO.

### Strengths and limitations

This study is based on self-reported information and recalled experience. However, drawing respondents from across different population groups and conducting FDGs across the community allowed researchers to triangulate the results and minimize potential bias. There is potential desirability bias among participants, particularly traditional healers. Two participants outside the desired inclusion criteria of the group ‘women of reproductive age’ participated in two of the FGDs—one in Panjane and one in Motaze. This did not result in exceptional responses during the respective FGDs and they had no apparent influence on overall responses from the concerned group. The two-stage translation process and the deployment of multiple interviewers were additional limitations of the study. Nevertheless, transcripts translations were conducted by certified local (Mozambican) translators and validated by in-house/local researchers. Interviewers were trained professionals and underwent workshops on the FGD materials used in this study.

## Conclusion

The residents of Magude showed relatively high awareness of malaria, trusted the health institutions, and identified gaps in prevention offered by the vector control tools. These favourable perceptions and behaviours emerged prior to the elimination campaign and may suggest the community’s openness to new intervention. However, a lack of awareness of asymptomatic carriers, inadequate understanding of residual transmission, and several barriers to seeking care might affect the overall strategy of malaria elimination in Magude. Continuous improvement of IEC is needed to emphasize the importance and benefits of the on-going control programmes (especially IPTp, bed net, and IRS) while integrating the concepts related to community-based chemoprophylaxis to maximize MDA uptake. Altogether, these findings suggest that malaria elimination efforts in southern Mozambique will strongly benefit from the intensified community engagement campaigns that reinforce prevention and treatment concepts while including messages to promote new interventions based on the local context.

## Additional file

**Additional file 1.** Tabulated summary of the key results from the Focus Group Discussions in each community group.

## Data Availability

All data are within the manuscript and the Additional file 1.

## Abbreviations

ACT: artemisinin-based combination therapy
AMETRAMO: Associação de Médicos Tradicionais de Moçambique
APE: Agentes Polivalentes Elementares
FGD: focus group discussion
HF: Health Facility
IPTp: Intermittent Preventive Treatment of Pregnant Women
IRS: indoor residual spraying
LLIN: long-lasting insecticidal nets
MDA: mass antimalarial drug administration
NMCP: National Malaria Control Program
RDT: rapid diagnostic test
UNFPA: United Nations Population Fund
WHO: World Health Organization
WMR: World Malaria Report
