# Supplementary material for: Examining community perceptions of malaria to inform elimination efforts in Southern Mozambique: a qualitative study
Source: Malar J. 2019 Jul 11;18:232. doi: 10.1186/s12936-019-2867-y (PMC6625114; doi:10.1186/s12936-019-2867-y)
Supplement: Supplementary file 1 — Additional file 1. Tabulated summary of the key results from the Focus Group Discussions in each community group. [file 12936_2019_2867_MOESM1_ESM.docx]

**SUPPLEMENTARY DATA**

**Title**

Examining community perceptions of malaria to inform elimination efforts in Southern Mozambique: a qualitative study

**Authors**

Harvie P. Portugaliza^1,2,3+^*, Beatriz Galatas^1,4+^, Hoticha Nhantumbo^4^, Helder Djive^4^, Ilda Murato^4^, Francisco Saúte^4^, Pedro Aide^4,5^, Christopher Pell^2^, Khátia Munguambe^4,6^

Affiliations

1. ISGlobal, Hospital Clínic - Universitat de Barcelona, 08036 Barcelona, Catalonia, Spain
2. Academic Medical Centre, University of Amsterdam, The Netherlands
3. Department of Biomedical Sciences, Institute of Tropical Medicine, 2000 Antwerp, Belgium
4. Centro de Investigação em Saúde da Manhiça (CISM), Manhiça, Mozambique
5. National Institute of Health, Ministry of Health, Maputo, Mozambique
6. Universidade Eduardo Mondlane, Maputo, Mozambique

*Correspondence: [harvie.portugaliza@isglobal.org](mailto:harvie.portugaliza@isglobal.org)

^+^Contributed equally

# Additional File 1: Tabulated summary of the key results from the Focus Group Discussions in each community group

**LIST of Tables**

- Table 1. List of Focus Group Discussions
- Table 2. Clinical signs and symptoms associated with different malaria terminologies mentioned by participants
- Table 3. Community perceptions of the cause of malaria
- Table 4. Community perceptions of malaria preventive measures
- Table 5. Malaria risk-taking behavior in Magude district

Table 1. List of Focus Group Discussions

| **Group** | **Location** | **Time** | **Participants** |
| --- | --- | --- | --- |
| **Adult men** | | | |
| FGD1_M16 | Magude Center | 38 min | 6 |
| FGD2_HA4 | Motaze | 90 min | 5 |
| FGD3_HA7 | Panjane | 58 min | 12 |
| FGD4_HA10 | Mahele | 97 min | 12 |
| FGD5_HA15 | Mapulanguene | 55 min | 4 |
| **Community leaders** | | | |
| FGD1_LC1 | Magude Center | 76 min | 7 |
| FGD1_LC5 | Magude Center | - | 3 |
| FGD2_LC3 | Motaze | 70 min | 12 |
| FGD3_LC8 | Panjane | 62 min | 12 |
| FGD4_LC11 | Mahele | 63 min | 10 |
| FGD5_LC14 | Mapulanguene | 55 min | 8 |
| **Women of reproductive age** | | | |
| FGD1_MIR6 | Magude Center | 43 min | 7 |
| FGD2_MIR2 | Motaze | 31 min | 12 |
| FGD3_MIR9 | Panjane | 45 min | 12 |
| FGD4_MIR12 | Mahele | 50 min | 12 |
| FGD5_MIR13 | Mapulanguene | 30 min | 10 |
| **Healer** | | | |
| FGD1_MT17 | Mixed location | 58 min | 10 |

Table 2. Clinical signs and symptoms associated with different malaria terminologies mentioned by participants

| **Participants** | **Malaria** | **Musothoto** | **Dze-dze-dze** |
| --- | --- | --- | --- |
| Community leader | Fever, headache, joint pain, weakness, cold, vomiting, back pain, confused thinking  Childhood malaria: severe stomachache, swollen belly, convulsion, weakness | Signs same with malaria | Vomiting, diarrhea, trembling |
| Adult Men | Weakness, headache, fever, shivering, chilly trembling, vomiting (*caguba*), stomachache, diarrhea, lack of appetite, afternoon cold, body pain | Headaches, body pain, diarrhea, vomiting, headache, disease of falling (“epilepsy”), joint pain, fevers, discouraged and unhappy, weakness, shivering, warm body, feel cold, wants to sleep, quiet (*Ku zinga*) | Vomiting, trembling, feel cold, binding of limbs, diarrhea, headaches |
| Women of Reproductive Age | Body pain, weakness, headache, joint pain,  fever, vomiting | Vomiting (bile) | Cold, vomiting bile, joint pain, chills, headaches, body pain |
| Healer | Fever, headache, body pain, cold, shivering, vomiting |  | Signs same with malaria |

Table 3. Community perceptions of the cause of malaria

| **Perceived cause of malaria** | **Community leaders** | **Adult men** | **Women of reproductive age** | **Healer** |
| --- | --- | --- | --- | --- |
| *Mosquito* | *Mosquito names*   - Female mosquito - Mosquito - Malaria mosquito   *Mosquito biting*   - Mosquito reproduces in water, enters houses and bites people - Mosquito bites dogs, snakes, and sick person, and then bites a healthy person. - Mosquito stings and litters the person with dirt   *Mosquito associated with water*   - Mosquito walks in ditches - Malaria mosquito from dirty water - Mosquito from stagnant water - Mosquito multiplies in streams - Mosquito reproduces in water, enters houses and bites people   *Mosquito associated with animals*   - Mosquito bites dogs, snakes, and sick person, and then bite a healthy person. - Mosquito proliferates in the presence of animals and animal pens   *Mosquito associated with plants*   - Mosquito from cane-field - Mosquito from grass around houses - Mosquito from pumpkin and watermelon   *Mosquito that transfers biological materials*   - Mosquito stings and litters the person with dirt - Mosquito transfers poison. - Mosquito has venom. | *Mosquito names*   - Mosquito - Anopheles   *Mosquito biting*   - Mosquito bites a person with malaria and bites another person - Mosquito bites an animal and bites a person - Mosquito eats dirt and bites a person - Mosquito bites when spraying agents have gone - Mosquito bite transfers blood from infected person - Mosquito that come into houses during nightfall to bite a person - Mosquito becomes the parasite that sucks on human blood   *Mosquito associated with water*   - Mosquito exists in abundance during rainy season - Mosquito reproduces in stagnant water or rain water. - Mosquito multiplies in the trash and dirty water   *Mosquito associated with animals*   - Mosquito bites an animal and bites a person   *Mosquito that transfers biological materials*   - Mosquito lands on dirt (e.g. feces) and then goes to a person to transfer the dirt - Mosquito eats dirt and bites a person - Mosquito bite transfers blood from infected person | *Mosquito names*   - Mosquito   *Mosquito biting*   - Mosquito bites a person with malaria and transmits to another person - Mosquito bites unprotected persons   *Mosquito associated with water*   - Mosquito in bathroom dirty water - Mosquito stays in dirty water   *Mosquito associated with hygiene*   - Mosquito comes when you leave trash in the house - Mosquito comes when we gather dirt or not take care of ourselves | *Mosquito names*   - Female mosquito   *Mosquito biting*   - Mosquito bites a person with malaria and then bites another person |
| *Others:*  *Dirty water, flies, insects, food, bad-smelling, virus, witchcraft and sorcery* | *Water*   - Drinking filthy water from Incomate river where person wash clothes - Drinking dirty water - Drinking river water with bacteria   *Flies and insects*   - Flies land on human feces and land on children food - Flies that land on peeled mango fruit during summer - Dirt at home - Bites from different insects   *Food*   - Eating uncooked food like salad and tomatoes causes malaria and cholera   *Bad-smelling*   - Inhaling “bad air” during defecation   *Malaria virus*  *Witchcraft and Sorcery* | *Water*   - Dirty water where children play - Water where mosquito develops - Dirty water used for cooking - Rains cause malaria - Drinking water from boreholes has mud and mosquito dirt   *Flies and insects*   - Flies at home due to lack of hygiene - Flies land on dirt (human feces) and land on food   *Food*   - Eating fresh food like pumpkins and coots   *Malaria virus*  *Witchcraft* |  | *Water*   - Water urinated by ox collected by a person - Littering the river - Drinking dirty water causes malaria or cholera   *Food*   - Eating dirty food - Eating plenty of watermelon   *Sorcery* |

Table 4. Community perceptions of malaria preventive measures

| **Community leaders** | **Adult men** | **Healers** | **Women of reproductive age** |
| --- | --- | --- | --- |
| **Vector-human contact avoidance** | | | |
| - Sleeping at night under the bed net - Child sleeps under the bed net - Burning insecticidal coils (Dragon and Baygon) - Closing windows at dusk and night - Personal hygiene and sanitation | - Sleeping at night under the bed net (Mutchiquitelo) - Burning insecticidal coils (Dragon and Baygon) - Personal hygiene and sanitation | - Personal hygiene and sanitation | - Sleeping at night under the bed net (Mutchiquitelo)   (only and most important way to avoid malaria-causing mosquito)   - Use of insect repellants - Use of coils |
| **Mosquito breeding and resting sites elimination** | | | |
| - Eliminate containers that retain water (e.g. bottles, coconut shell [cafulo]) - Eliminate stagnant water from old tires - Clean the water pits in the bathroom - Eliminate trash - Burn and burry trash - Pruning trees - Avoid pile of clothes - Clean houses - Move animal pens far from houses - Cleaning children feces - IRS   -scare away mosquito (children don’t know to scare mosquito away)  -kill mosquito | - Eliminate puddle of water - Eliminate dirty water - Throw empty cans properly - Medicine to treat ponds (tlhive) and lagoons against mosquito (you cannot cover the ponds) - Clean and cover water holes and wells to avoid dirt (e.g. tree leaves) - Cover the pits to avoid rain water from entering and rotting that will attract mosquito breeding - Weed plants in the backyard - Avoid hoarding garbage - Eliminate trash - Clean yards - Clean house and bathroom | - Eliminate water from rainwater bowls - Remove rainwater from tires and cisterns - Eliminate stagnant water - Prune trees - Cleaning homes - Clean dropped tree leaves - IRS   -scare away mosquito | - Remove dirty water - Cleaning homes - Eliminate holes with water - Burn trashes - Water far from houses - Prune trees and shrubs - Clean houses - Eliminate trash - Burry and burn dirt - IRS |
| **Chemoprophylaxis or disruption of symptoms initiation** | | | |
| - Protective pills   -Avoid mosquito bites  -Way of Immunization  -Prevent mosquito from transmitting malaria  - Avoid getting sick when mosquito bites | - Protective pills   - Avoid getting sick when mosquito bites  -Intermittent preventive treatment for pregnant |  | - Protective pills   - perceived to mainly avoid mosquito bites |
| **Other measures of prevention** | | | |
| - Hygiene and sanitation related to toilet and defecation. - Clean air to breath by cleaning houses - Hand washing | - Hygiene and sanitation related to toilet and defecation - Drinking clean water |  |  |

Table 5. Malaria risk-taking behavior in Magude district

| **Community leaders** | **Adult men** | **Healers** | **Women of reproductive age** |
| --- | --- | --- | --- |
| **Indoor malaria risk-taking behavior and activities** | | | |
| - Not using bed net   -Still in plastic   - Misusing bed net   -use for fishing   - Refusing IRS   -causes cough  -cockroaches appear  -have air-con.  -house can get dirty   - Not closing windows at night | - Not using bed net   -Cannot sleep   - Misusing bed net   -use for fishing   - Refusing IRS | - Misusing bed net   -use to encircle cabbage | - Not using bed net - Sitting in common areas - Sleeping late - Not using insect repellant and coil |
| **Outdoor malaria risk-taking behavior and activities** | | | |
| - Defecating in the bushes - Visiting and near cane plantation - Going to the forest to look for roots (herbal medicine) - Harvesting fruits and vegetables - Fetching drinking water from the river - Chatting in the backyard - Passing at garbage site | - Bathroom and toilet in the bushes - Harvesting fruits and vegetables - Children play in water areas - Near animal barn |  | - Attending night class - Diurnal mosquito biting - Not using insect repellant |
